# The complete chloroplast genome of *Lagerstroemia balansae*, an endangered species of genus *Lagerstroemia* native to China

**DOI:** 10.1080/23802359.2021.1882352

**Published:** 2021-03-01

**Authors:** Bo Qin, Kaidao Sun, Xin Huang

**Affiliations:** Guangxi Forestry Research Institute, Guangxi Key Laboratory of Special Non-wood Forest Cultivation & Utilization, Nanning, China

**Keywords:** Chloroplast genome, endangered species, *Lagerstroemia balansae*, phylogenetic analysis

## Abstract

We announce here the first complete chloroplast genome sequence of *Lagerstroemia balansae*, a plant species with extremely small populations rated the level of EN (Endangered) in China. This complete chloroplast genome is 152316 bp in size. In total, 130 genes were identified, including 85 protein-coding genes, 37 tRNA genes, 8 rRNA genes. The result of phylogenetic analysis strongly supported that *L. balansae* was closely related to *L*. *tomentosa*.

*Lagerstroemia* (Lythraceae) mainly distributed in the East and Southeast Asia, Southern and Northern Australia, with about 55 species worldwide (Furtado and Srisuko 1969; Qin and Graham 2007). Most of the species of the genus *Lagerstroemia* has a high ornamental value, which is widely used in tropical and subtropical regions in landscaping. *L. balansae* is a deciduous tree with yellow tomentose calyx, long-lasting lilac flowers and mottled green trunk, giving this species great ornamental value. *L. balansae* mainly distributed in Hainan province and Guangdong province, China. According to the Threatened Species List of China’s Higher Plants (Qin and Zhao 2017), it is rated the level of EN (Endangered). Giving the increasing threat to survival, conservation efforts are needed to protect the germplasm resources of *L. balansae*.

Molecular identification of *Lagerstroemia* cultivars and interspecific hybrids has been reported, but there is a lack of complete genome research on *Lagerstroemia* (Gu et al. 2016), with only 14 of 55 of the chloroplast genome belonging to *Lagerstroemia* have been available in NCBI GenBank. These species are as follows: *L. speciosa*, *L. tomentosa*, *L. venusta*, *L. fauriei*, *L. indica*, *L. subcostata*, *L. intermedia*, *L. floribunda*, *L. guilinensis*, *L. calyculata*, *L. excelsa*, *L. villosa*, *L. siamica*, *L. limii* (https://www.ncbi.nlm.nih.gov/nuccore/?term=Lagerstroemia). Chloroplast genomes are highly conserved and differ from nuclear genes in the rate of evolution. It is feasible and effective to study the evolution of genome structure and function of a species by using the whole chloroplast genome sequencing. We reported the complete chloroplast genome sequence of *L. balansae*, and the annotated genomic sequence was submitted to GenBank under the accession number MT942707.

The fresh leaves of *L. balansae* was collected from Guangxi University (Nanning, Guangxi, China; 22.83°N, 108.31°E), which previously introduced from Haikou (Hainan, China; 20.02°N, 110.11°E). Voucher specimen were deposited at the herbarium of Guangxi Forestry Research Institute (accession number: 2020080101), and DNA samples were stored at Guangxi Key Laboratory of Special Non-wood Forest Cultivation & Utilization, Nanning, China. 6.0 GB of raw data were generated with 150 bp paired-end read lengths, then the Get Organelle (Jin et al. 2018), Bandage (Wick et al. 2015), GeSeq (Tillich et al. 2017), were used to align, assemble, and annotate the chloroplast genome. The full length of *L. balansae* chloroplast genome was 152316 bp and comprised of a large single copy region (LSC with 84,053 bp), a small single copy region (SSC with 16,785 bp), and two inverted repeat regions (IR with 25,739 bp). The overall GC content of *L. balansae* complete chloroplast genome was 37.6%. A total of 130 genes were contained in the chloroplast genome, including 85 protein-coding genes, 37 tRNA genes and 8 rRNA genes.

To confirm the phylogenetic position of *L. balansae*, the complete chloroplast genome of *L. balansae* was aligned with 18 other species obtained from GenBank. Three species, i.e. *Cinnamomum camphora, Citrus reticulata and Vitis vinifera* cultivar *Maxxa* were used as an out group. All of these 19 complete chloroplast sequences were aligned by the MAFFT version 7.429 software (Katoh and Standley 2013). A maximum-likelihood (ML) tree was inferred by ultrafast bootstrapping with 1000 replicates through IQ-TREE 1.6.12 (Nguyen et al. 2015) based on the TVM + F+R2 nucleotide substitution model, which was selected by ModelFinder (Kalyaanamoorthy et al. 2017). The result of phylogenetic analysis strongly supported that *L. balansae* was closely related to *L. tomentosa* (Figure 1).

**Figure 1.**
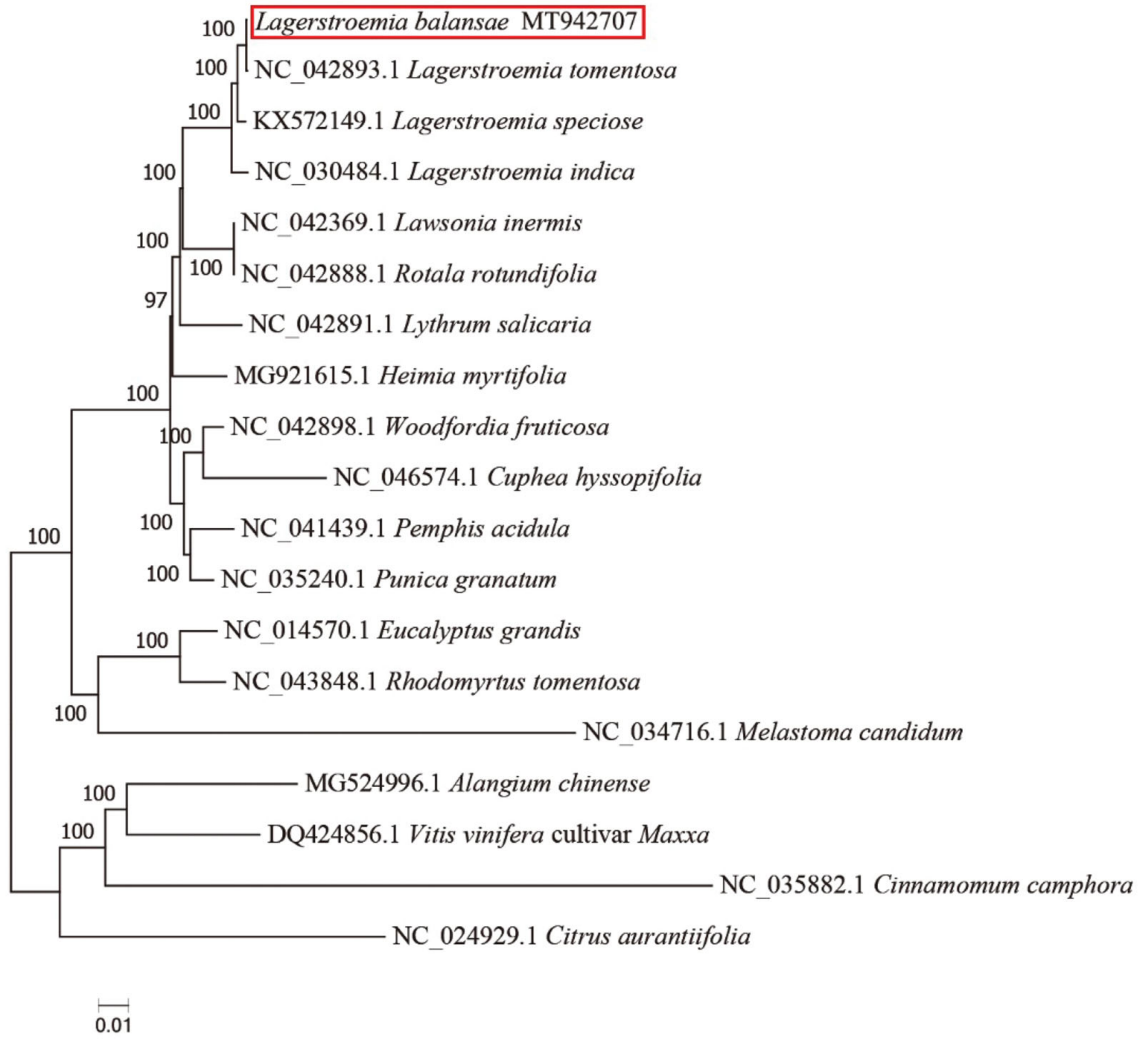
The ML phylogeny result from eighteen complete plastome sequences by IQ-TREE. Accession numbers: *Lagerstroemia tomentosa*: NC_042893.1; *L. speciose*: KX572149.1; *L. indica*: NC_030484.1; *Lawsonia inermis*: NC_042369.1; *Rotala rotundifolia*: NC_042888.1; *Lythrum salicaria*: NC_042891.1; *Heimia myrtifolia*: MG921615.1; *Woodfordia fruticose*: NC_042898.1; *Cuphea hyssopifolia*: NC_046574.1; *Pemphis acidula*: NC_041439.1; *Punica granatum*: NC_035240.1; *Eucalyptus grandis*: NC_014570.1; *Rhodomyrtus tomentosa*: NC_043848.1; *Melastoma candidum*: NC_034716.1; *Alangium chinense*: MG524996.1; *Vitis vinifera* cultivar *Maxxa*: DQ424856.1; *Cinnamomum camphora*: NC_035882.1; *Citrus aurantiifolia*: NC_024929.1.

## Data Availability

The data that support the findings of this study are openly available in GenBank number MT942707 (https://www.ncbi.nlm.nih.gov/nuccore/MT942707/) and SRA number PRJNA673929 (https://www.ncbi.nlm.nih.gov/sra/?term=PRJNA673929).
